# A Big Five-Based Multimethod Social and Emotional Skills Assessment: The Mosaic™ by ACT^®^ Social Emotional Learning Assessment

**DOI:** 10.3390/jintelligence10040072

**Published:** 2022-09-20

**Authors:** Kate E. Walton, Jeremy Burrus, Dana Murano, Cristina Anguiano-Carrasco, Jason Way, Richard D. Roberts

**Affiliations:** 1 ACT, Inc., Iowa City, IA 52243, USA; 2 Research and Assessment Design (RAD) Science Solution, Philadelphia, PA 19146, USA

**Keywords:** SEL, social and emotional learning, social and emotional skills, assessment, Big Five, MTMM, multi-trait multi-method

## Abstract

A focus on implementing social and emotional (SE) learning into curricula continues to gain popularity in K-12 educational contexts at the policy and practitioner levels. As it continues to be elevated in educational discourse, it becomes increasingly clear that it is important to have reliable, validated measures of students’ SE skills. Here we argue that framework and design are additional important considerations for the development and selection of SE skill assessments. We report the reliability and validity evidence for The Mosaic™ by ACT^®^ Social Emotional Learning Assessment, an assessment designed to measure SE skills in middle and high school students that makes use of a research-based framework (the Big Five) and a multi-method approach (three item types including Likert, forced choice, and situational judgment tests). Here, we provide the results from data collected from more than 33,000 students who completed the assessment and for whom we have data on various outcome measures. We examined the validity evidence for the individual item types and the aggregate scores based on those three. Our findings support the contribution of multi-method assessment and an aggregate score. We discuss the ways the field can benefit from this or similarly designed assessments and discuss how the assessment results can be used by practitioners to promote programs aimed at stimulating students’ personal growth.

## 1. Introduction

In the past 25 years, social and emotional learning (SEL) has demonstrated tremendous growth and continues to gain popularity. There is growing consensus that a number of factors outside of cognitive ability may be nearly, or just, as important for educational and workplace success (e.g., Aspen Institute 2018; Burrus et al. 2017; Durlak et al. 2015; Kautz et al. 2014). These factors are often termed social and emotional (SE) skills, which can be defined as, “individual capacities that (a) are manifested in consistent patterns of thoughts, feelings, and behaviours, (b) can be developed through formal and informal learning experiences, and (c) influence important socioeconomic outcomes throughout the individual’s life” (OECD 2015, p. 34). SE skills predict a variety of important outcomes, which include, but are not limited to, health (Bogg and Roberts 2004), academic performance (Chernyshenko et al. 2018; Mammadov 2021; Poropat 2009), and job performance (Barrick et al. 2001; Zell and Lesick 2021).

As a result of this growing consensus, the implementation of SEL curricula outpaces the development of assessments through which SE skills can be measured (e.g., Denham 2015). Clearly, reliable and valid measures of students’ SE skills are key for tracking student growth and for studying intervention efficacy. Beyond the evidence for reliability and validity, we encourage developers and users to consider two additional features of the SE skill measures—the framework on which the measure is based and the nature of the items that comprise the measure. Though a vast amount of SE skill frameworks exist (see Jones et al. 2016), some frameworks enjoy more empirical backing than others. Likewise, some item types may be more or less advantageous in certain situations. Here, we discuss one particular SE skills assessment, The Mosaic™ by ACT^®^ Social Emotional Learning Assessment (formerly known as ACT^®^ Tessera^®^ and referred to as Mosaic in the remainder of this paper), as an example of an assessment that makes use of an empirically backed framework and a multimethod approach.

## 2. The Big Five as an Organizing Framework for SE Skills

The Big Five personality trait model is guided by the lexical hypothesis, which assumes that important individual differences will become encoded into language as single terms (Goldberg 1993). The model was born after researchers turned to an English language dictionary and used factor analytic techniques (e.g., Cattell 1943; Tupes and Christal 1961) to uncover five replicable factors. These factors are commonly labeled Extraversion, Agreeableness, Conscientiousness, Emotional Stability, and Openness to Experience, and are often referred to as the Big Five (de Raad and Mlačić 2015). Subsequently, the same five factors have been found in other languages using various methods (e.g., McCrae et al. 2005; Schmitt et al. 2007).

Conceptually, the Big Five can be considered a “Rosetta Stone” for understanding SE skills (Martin et al. 2019). Using the Big Five, we can take constructs expressed as time management in one framework and grit in another, and understand their connectedness as components of Conscientiousness. Soto and colleagues (2022) provide a comprehensive model of SE skills that resembles the Big Five and have constructed a psychometrically sound SE skill inventory based on this model. Additional recent work speaks to the empirical merit of the Big Five-SE skill links. For example, Walton and colleagues (2021) carried out two studies using different methodologies to evaluate this. In one, they asked subject matter experts from the fields of personality psychology and SEL to rate the degree of overlap between the Big Five traits and popular SE skills. As anticipated, the traits and skills were deemed to overlap to a significant degree. In another study, they factor analyzed items from a Big Five measure along with items from a CASEL (the most prominent SEL framework in the US) competency-based measure, and they determined that the best fitting model was one with five factors that aligned with the Big Five traits.

The Big Five is also an ideal candidate for SE skills assessments because of its consistency with SE skills definitions. Recall that the OECD’s (2015) definition states that SE skills influence important outcomes. There is a vast body of research linking the Big Five with many critical outcomes. For example, a meta-analysis by Poropat (2009) found that, during the primary school years, the cognitive ability’s impact on academic performance exceeds that of any Big Five trait, but by secondary education, Conscientiousness is nearly as important for academic performance as cognitive ability. Another recent meta-analysis also found that Conscientiousness was a strong predictor of academic performance across all levels of schooling and predicted performance incrementally above-and-beyond cognitive ability (Mammadov 2021).

As another example of why the five-factor model is a good choice of framework for SE skills, the OECD’s (2015) definition states that such skills can be developed. In their meta-analysis of mean-level personality change, Roberts et al. (2006) found that individuals exhibit development throughout their lifespan. Furthermore, meta-analyses demonstrate that interventions can alter personality traits significantly even when the interventions last for short periods of time (Hudson et al. 2019; Roberts et al. 2017). Similarly, meta-analyses speak to the effectiveness of SEL interventions. Students who participate in SEL programs see greater gains in SE skills and academic performance versus students who do not participate, and both short- and long-term benefits have since been documented (e.g., Corcoran et al. 2018; Mahoney et al. 2018).

Due to its utility in conceptualizing SE skills and growing empirical support in the field, the Big Five has been adopted as an organizing framework for SE skills in prior literature (Abrahams et al. 2019; Casillas et al. 2015; Soto et al. 2022) and other work. Notably, the OECD’s Study on Social and Emotional Skills, which is a large-scale international study, uses the framework (Chernyshenko et al. 2018; Kankaraš and Suarez-Alvarez 2019).

## 3. Methodological Approaches to the Assessment of Social and Emotional Skills

Several item types have been used to assess SE skills. For example, Likert items have been used in SEL research for decades and are known for their efficiency, reliability, and validity. Individuals indicate their agreement with several statements (e.g., “*I work hard*”). This item type is preferred when stakes are low and faking is not expected (Lipnevich et al. 2013). However, respondents may have various motives to fake, such as appearing more attractive to a school admissions officer (Zickar et al. 2004). Furthermore, Likert items might be particularly susceptible to reference effects. That is, people often answer such items by asking the question, “compared with whom?” For example, it could be the case that students from very high achieving schools might rate themselves lower on their SE skills than students from low achieving schools simply because they are using a different reference group, not because they are truly lower on these skills (e.g., Marsh and Hau 2003).

Forced choice (FC) items have been used to assess individuals’ tendencies and capabilities as well. With FC items, statements are grouped in blocks and respondents make selections within each block regarding which statements describe him or her best. There are several variations of the FC methodology (Hontangas et al. 2015). For example, respondents may read multidimensional triads and choose, of the three items (e.g., “*I enjoy leading class discussions*”, “*I work hard in school to achieve my goals*”, and “*I like working in a team*”), which is most like them and which is least like them. Reference bias should be minimized with FC because respondents conduct an internal (self vs. self) rather than an external (self vs. other) comparison on these items. Furthermore, there are compelling studies (Christiansen et al. 2005; Jackson et al. 2000; Walton et al. 2021) and meta-analytic data (Cao and Drasgow 2019) suggesting that FC items cannot be faked as easily as Likert items. For example, Christiansen et al. studied responses across two conditions, one in which participants were instructed to answer honestly and one in which participants were instructed to answer as if they were applying for a job and trying to make a positive impression. The effect size (i.e., standardized mean difference in the responses across the honest and positive impression conditions) was just .24 for the FC items, but the effect reached .96 for the single stimulus items.

A third item type used in this domain is the situational judgment test (SJT). In this approach, students imagine themselves in a scenario and then read various behavioral responses to that scenario. They then indicate either how likely they *would* be to respond in that manner or are asked to select how a person *should* respond in that scenario. SJTs have several advantages. First, SJTs may be developed to reflect more complex judgment processes than what is possible with Likert scales. Second, SJTs have the advantage of face validity (Lievens et al. 2008). The situations presented to students have the look and feel of those that would be encountered in real life. Third, there is evidence suggesting that SJTs are less prone to faking than Likert items (Hooper et al. 2006), and some have argued for using them in high-stakes settings for this reason (Whetzel and McDaniel 2016). Fourth, SJTs should be less susceptible to reference effects than Likert items because behavioral responses to scenarios are not comparative in nature. They have been used for decades for employee selection, and now even the Association of American Medical Colleges (2022) has developed an SJT measure to assess medical students’ knowledge about effective behaviors.

Given that all item types have different strengths and weaknesses, an assessment employing multiple methods (i.e., one that conforms to a *multi-trait multi-method* [MTMM] design) ought to minimize the effects of any biases or shortcomings. According to Kenny and Kashy (1992), “The underlying view of measurement in the MTMM analysis is that to measure a theoretical construct, different measures, each with its own bias, are selected. Bias that is due to method effects is reduced through a triangulation process” (p. 170). To our knowledge, there is no research specifically examining the validity evidence for a MTMM SE skills assessment. It is worth examining whether the inclusion of varied item types contributes to the prediction of important outcomes.

## 4. Current Study

In this study, we briefly describe Mosaic, a multi-method SE skills assessment based on the Big Five framework. In addition to determining the extent to which the Big Five can account for the variance in important school outcomes, we sought to examine whether Mosaic’s MTMM approach leads to stronger validity evidence than what would be available from the individual item types. We anticipated our findings could help inform framework and methodological considerations for future SE skills assessments.

## 5. Method

### Participants

During the 2018–2019 academic year, 24,400 students from 160 schools completed Mosaic Middle School (MS). The demographic information was provided by the schools. The grade level breakdown was: 6th = 3864 (15.8%), 7th = 17,585 (72.1%), and 8th = 2951 (12.1%). The gender breakdown was: female = 12,273 (50.3%) and male = 12,127 (49.7%). Nine thousand one hundred twelve students from 93 schools completed Mosaic High School (HS). The grade level breakdown was: 9th = 5413 (59.4%), 10th = 1739 (19.1%), 11th = 1002 (11.0%), and 12th = 958 (10.5%). The gender breakdown was: female = 4792 (52.6%) and male = 4320 (47.4%). Race/ethnicity data were not available for most participants, and SES is not available for any students. Some data were missing for some schools or students so not all *N*s are identical across the measures.

The students were enrolled in schools that purchased the assessment as part of their SEL programming; that is, the students completed the assessment as part of their regular school activities and were not solicited for participation in the research. Students gave their assent before completing the assessment by checking “yes” to an item worded “By clicking YES, I assent to participate in this activity”. The schools were spread across the U.S. and were a mixture of public, private, and charter schools. Schools allotted one class period for completion and most students were able to complete the assessment during that time but were able to take longer than one class period if necessary.

## 6. Measures

### 6.1. Mosaic™ by ACT^®^ Social Emotional Learning Assessment

**Constructs.** The SE skills assessed by Mosaic (ACT 2021) can be aligned with the Big Five constructs on a one-to-one basis (Table 1). This alignment was conducted rationally by subject matter experts comparing the Big Five factors’ definitions and Mosaic item content and skill definitions. The Mosaic skill labels are different from the Big Five trait labels in accordance with educator preference. For example, focus group feedback indicated that the term Social Connection is preferred over Extraversion. The different labels notwithstanding, the construct breadth is similar to the Big Five. For example, the Social Connection items measure more than that single facet of Extraversion; they also tap into assertiveness, leadership, persuasiveness, etc.

**Item Types.** Mosaic employs the MTMM approach discussed above with Likert, FC, and SJT items. An example Likert item measuring Getting Along with Others is: *I offer to help those who need assistance*. Students indicate their level of agreement with this statement on a six-point scale from *Strongly Disagree* to *Strongly Agree*. There are 40 Likert items with eight per construct. The descriptive statistics and reliability estimates, all of which exceed .70 for MS and HS forms, can be found in Table 2.

Below is an example of a multidimensional FC triad. Students are instructed to select the statement that is most like them and the one that is least like them. In the example below, the statements measure, respectively, Keeping an Open Mind, Sustaining Effort, and Getting Along with Others.

- *I perform well on assignments that require me to use my imagination to find the answers.*
- *If I tell my teacher I will do something, I do it.*
- *I ignore classmates who are being left out of class discussions.*

There are $\left(\begin{array}{c} 5\\ 3 \end{array}\right)=$10 FC triads. Each possible triad occurred once and only once, so there are a total of 30 items with six per construct. Cronbach’s alpha values (see Table 2) were lower than what is typically found with Likert items, which is to be expected given their ipsative nature (Meade 2004).

Mosaic includes two SJTs per construct yielding 10 total items. Again, Cronbach’s alpha values (see Table 2) were lower than what is typically found with Likert items, given that they are often multidimensional (Whetzel and McDaniel 2009). Students read each scenario then are presented with five behavioral responses, all of which measure a single construct, and they are asked to indicate how likely they are to enact each response on a five-point scale from *Very Unlikely* to *Very Likely*. An example of a partial SJT measuring Sustaining Effort reads:

*After studying very hard for a math test, the test results *are* disappointing, and you have yet to do as well as expected. While you are currently proficient, you would like to move up to the next level.*

Two of the behavioral responses read:

- *Look over the test to see what questions you got wrong and work on those.*
- *Decide there’s no point to studying so hard if you don’t get the results you want.*

**Scoring.** Negatively worded items across the three item types were reverse scored. The Likert and SJT items were scored in the typical manner by averaging across all responses to yield a total score per construct where higher scores are aligned with higher skill levels. The FC items were scored such that the statement that is selected as ‘most like me’ was scored with a 3, the statement not selected was scored with a 2, and the statement selected as ‘least like me’ was scored with a 1, then the values were averaged across the responses to yield a total score per construct where higher scores were aligned with higher skill levels.

To create aggregate scores across the three item types, the Likert, FC, and SJT scores were standardized then averaged. It is important to note that it is strongly preferred to derive a single score per skill for practical purposes. When reporting SE skill scores to students, parents, and educators, this is a more parsimonious approach rather than reporting three scores per skill. Thus, it is not our goal to examine the incremental validity of the item types; that is, below we compare the validity evidence for the single item types and aggregate scores, rather than building a hierarchical model and examining the incremental validity.

### 6.2. Test-Criterion Data

Below we describe each of the outcome variables that we obtained from the sub-samples of the 33,512 students completing either Mosaic MS or HS.

**School Climate.** To measure school climate, we used the Relationships with School Personnel and School Safety Climate (hereafter referred to as Relationships and Safety, respectively) scales from ACT Engage^®^. The scales are supported by strong evidence of reliability and validity (ACT 2016) and have high internal consistency estimates. There were 12 Relationship climate items (Cronbach’s alpha MS = .86 and HS = .88) and 11 Safety climate items (Cronbach’s alpha MS = .84 and HS = .85), all of which are Likert items rated on a six-point agreement scale. These items were identical across the two forms. All Relationships items make mention of how a student perceives his or her relationships with adults at school (e.g., *There are adults at my school who care about me*). The Safety items primarily focus on students’ perceptions of their physical safety in school (e.g., *I feel safe at school*). The climate scores used in the current study reflect each student’s individual perceptions. Prior research has shown that SE skills and favorable perceptions of school climate are positively related to one another (Allen et al. 2019; Osher and Berg 2017).

**Academic Performance.** GPA was self-reported on a 12-point scale (e.g., A+, 97–100%; A, 93–96%; etc.). Meta-analytic data (Mammadov 2021; Poropat 2009) indicate that the Sustaining Effort skill is expected to have the strongest relationship with GPA.

**Attendance.** Four school districts provided additional MS student data, including absences and disciplinary infractions, and 12 districts reported these data for HS students. We examined the association between Mosaic scores and absenteeism. We used the sum of unexcused and excused absences as a continuous variable and, when appropriate, also split the sample into three groups representing students with chronic absenteeism (defined as 18 or more missed days), habitual absenteeism (defined as at least 10 missed days but fewer than 18), or acceptable absenteeism (fewer than 10 missed days) and examined group mean differences. Most states describe chronic absenteeism as missing 10 percent or more days within a year (Attendance Works n.d.), which equates to 18 or more days, while some states consider missing 10 or more days within a school year as being habitually truant (Colorado Department of Education n.d.). Based on the findings reported by Lounsbury et al. (2004), we expected Keeping an Open Mind to have the strongest association with absenteeism and that they would be negatively related. However, the opposite effects have been found in college students (Chamorro-Premuzic and Furnham 2003; Credé et al. 2010), so this is somewhat of an exploratory analysis.

**Discipline.** Finally, we considered the number of disciplinary infractions as recorded in school records. For some schools, we had a count of the actual number of disciplinary infractions, but in some cases, schools only provided a binary response (i.e., no infractions vs. at least one). For some analyses, we dichotomized the continuous responses that some schools had provided and combined them with the binary responses. Research on children and adolescents (Tackett 2006; Tackett et al. 2013) led us to anticipate negative associations between disciplinary infractions and Sustaining Effort, Getting Along with Others, and Maintaining Composure. We anticipated positive, yet smaller, associations between the disciplinary infractions and Keeping an Open Mind and Social Connection.

## 7. Results

### 7.1. Convergent and Discriminant Validity

The complete MTMM correlation matrix can be found in Table 2. There is evidence for convergent validity with the Likert scales generally correlating most highly with their respective SJT and FC scales and the SJT scales correlating most highly with their respective FC scales. For example, in MS, the correlation between the Likert Maintaining Composure scores and the SJT Maintaining Composure scores (*r* = .54) was higher than between the Likert Maintaining Composure scores and any of the other four SJT scores (*r* = .28–.47). However, evidence for discriminant validity was not as strong, as there were several instances of off-trait correlations being higher than expected. For example, in MS, the correlation between the Likert Sustaining Effort scores and the SJT Maintaining Composure scores reached .59. There were fewer instances of this occurring in HS than in MS.

### 7.2. Test-Criterion Validity

For the MS and HS forms, we examined the test-criterion validity evidence by correlating the SE skills with the outcome measures. We also regressed the outcomes on the SE skill scales. We did this for each item type individually and compared the findings to those for the aggregate skill scores.

#### 7.2.1. School Climate

As expected, all correlations between SE skills and school climate were positive (Table 3). MS associations were slightly stronger than those observed in HS, and SE skills had slightly higher correlations with Relationships than with Safety. For both MS and HS forms and both climate scales, either the Likert or the aggregate scores had the strongest correlations. The Likert scales (MS Relationships *R*^2^ = .38; MS Safety *R*^2^ = .24; HS Relationships *R*^2^ = .28; HS Safety *R*^2^ = .16) accounted for slightly more variance than the aggregate sores (MS Relationships *R*^2^ = .36; MS Safety *R*^2^ = .22; HS Relationships *R*^2^ = .25; HS Safety *R*^2^ = .14).

#### 7.2.2. Academic Performance

For both forms, Sustaining Effort had the strongest relationship with GPA (Table 3). However, we should note that all skills were related to academic performance, a finding not typically reported in prior research (Mammadov 2021; Poropat 2009). We suspect this is due to the fact that many of our items are contextualized to a school setting, which may lead to higher correlations than what is found with “generic” Big Five measures. Across skills, the aggregate scores generally had the strongest correlations with GPA. In MS and HS, the Likert (MS *R*^2^ = .17; HS *R*^2^ = .23) and aggregate scores (MS *R*^2^ = .18; HS *R*^2^ = .23) were on par with one another in terms of variance in GPA accounted for.

#### 7.2.3. Attendance

We examined correlations between the number of absences and the five SE skills (Table 3). In MS, the correlations were all near zero and there was no clear pattern in terms of which item type exhibited the strongest association. In HS, the correlations were generally small and negative, and the SJTs or aggregate scores had the strongest associations. The individual item types and the aggregates did not count for much variance in absenteeism (i.e., no more than 4%). To evaluate whether the effects varied across item types and aggregate scores, we split the HS sample into three groups—students with chronic, habitual, or acceptable absentee records. All effect sizes were small, though the SJT and aggregate effect sizes were slightly higher overall (Table 4).

#### 7.2.4. Discipline

We examined the correlations between the number of reported disciplinary infractions and the five SE skills (Table 3). In MS, the correlations were in the expected direction. In HS, the Social Connection correlation was higher than expected, relative to those for Sustaining Effort, Getting Along with Others, and Maintaining Composure. The SJT and aggregate associations were generally the strongest. In MS, the SJTs accounted for 12% of the variance in disciplinary infractions, while the aggregate scores accounted for 9%. In HS, however, the individual item types and the aggregate scores were fairly even in terms of variance accounted for, with values ranging from 4% to 6%.

As previously stated, some schools only provided a binary response (i.e., no infractions vs. at least one). We dichotomized the continuous responses that some schools had provided and combined them with the binary responses and carried out independent samples *t*-tests to compare these students and determine if results differed across item type. Results are reported in Table 5. Any differences in the direction of the effect across the binary and continuous analyses are due to non-identical samples used across the analyses. In MS, the SJT and aggregate scores had the greatest effect sizes. The results were more varied in HS, but generally the SJTs had the greatest effect sizes.

## 8. Discussion

The MS and HS Mosaic forms were developed using the Big Five as its assessment framework and a multi-method approach to measure SE skills. We primarily focused on the validity evidence for three item types (Likert, FC, and SJT) and an aggregate score based on these three. Overall, the associations between the five SE skills, regardless of item type, and the outcome measures were as expected. For example, all skills were positively associated with school climate dimensions. We found support for the use of an aggregate score and determined that, depending on the particular outcome of interest, certain item types may provide stronger test-criterion validity evidence.

### 8.1. Methodological Considerations

The Likert item scores and aggregate scores were the best predictors of perceptions of school climate and GPA. The SJT-based and aggregate scores showed the strongest effects in terms of attendance and discipline, although effects overall were smaller than what we observed for climate and GPA. The FC items failed to prevail as the strongest predictor for any of the outcomes we examined, though they were significant predictors of all outcomes except for attendance in MS. Note that unlike HS, there was no evidence of relationships between SE skills and absenteeism in MS, which likely reflects a greater autonomy among HS students relative to MS students.

It is not surprising that Likert items accounted for more variance in perceptions of school climate. The climate items were also Likert items and therefore the shared method variance is likely at play here. We can conjecture as to why SJTs had the strongest link with school attendance and discipline. As discussed above, SJTs have the “look and feel” of situations encountered in real life. Of the five outcomes we assessed, attendance and discipline are the most behavioral. That is, you attend school or you do not, and you get in trouble or you do not. These types of behaviors are written into SJTs. For example, one Mosaic SJT provides the possible behavior response of “*Angrily confront your coach…*” This is an example of a behavior that would likely lead to some disciplinary action. GPA is not as explicitly behavioral. There are multiple processes involved that lead to an academic measure and it’s a more distal outcome. That is, GPA stems from a combination of behavior, cognitive ability, course taking, and teacher judgment. As such, it’s not as pure as a behavioral measure. This may explain why the Likert items did a better job explaining the variation in student academic performance. As mentioned above, although significant predictors of outcomes, the FC items were not “the best” in terms of predicting the outcomes. This may be due to the low reliability of the scores, which, again, is typical for ipsative FC scores (Meade 2004). Despite this artefact of FC scores, FC items have other advantages, such as making it difficult for respondents to engage in impression management (Cao and Drasgow 2019), which might be particularly attractive in high-stakes settings.

In addition to the possibility of reducing the impression management with the FC items and the strong face validity of SJTs, there are other benefits to using a multi-method approach. As discussed above, reference effects and other response tendencies, such as acquiescent responding, might be minimized by using alternatives to Likert items. Another factor that has gone unmentioned until this point is the increased level of engagement respondents may experience when encountering more than one item type in an assessment. All of these factors, along with the evidence for improved validity from the current study, reinforce the notion that a multi-method approach to SE skills assessments has merit.

Many other assessments rely solely on Likert items to obtain scores (e.g., Davidson et al. 2018; LeBuffe et al. 2018). We believe the review and findings presented here provide compelling evidence supporting the inclusion of multiple item types and aggregate scores. This approach is novel, but it is slowly being adopted by others in the field. For example, the OECD’s Study on Social and Emotional Skills also uses an MTMM approach to generate SE skill scores (Kankaraš et al. 2019). In contrast to our approach, which involves multiple student-report item types, the OECD study combined Likert scales from parents, teachers, and students.

### 8.2. Framework Considerations

Mosaic differs from many SE skills assessments in terms of its organizing framework. This assessment makes use of the Big Five framework, which is advantageous because it is evidence-based, has cross-cultural generalizability, and consistently replicates across age groups. In addition, it can be used as a framework through which to organize the various terms used to describe SE skills (Walton et al. 2021). One issue plaguing the field of SEL is the abundance of frameworks currently used to organize skills, with a recent study identifying 136 frameworks (Berg et al. 2017). Moving toward a single assessment framework can help to unify the field and integrate research findings across research groups. A unified framework should be comprehensive yet parsimonious, consider developmental implications, and be evidence based and data driven, rather than derived from theory or expert consensus alone. The Big Five stands out as the organizing framework that fits each of these recommendations.

Despite support for using the Big Five framework to organize SE skills, it is imperative to recognize that personality traits and SE skills are not synonymous with one another. Once again, consider the OECD’s (2015) definition; it refers to SE skills as “individual capacities.” Capacities and tendencies are distinct, and herein lies the key differentiator between SE skills and personality traits. Soto and colleagues (e.g., 2021) are careful to make this point. While they use the Big Five framework for their assessment of social, emotional, and behavior skills, the BESSI, they reframe the item stems to ask respondents to report how well they are able to do certain things rather than how likely or how often they do those things (e.g., *How well can you perform in a leadership role? vs. How likely are you to take on a leadership role?*; Soto et al. 2022).

### 8.3. Applications for Educational Contexts

Research suggests that SE skills predict an array of desirable outcomes including success in school. Obtaining measures of these skills is key to fostering school-wide SEL initiatives. Practitioners can use data from SE skill assessments as part of a formative assessment system. Assessments can be considered formative if the feedback from the assessment is used to guide practices or make changes that increase student learning (Black and Wiliam 2009). An assessment itself is not necessarily formative on its own, but the process of a formative assessment entails multiple learning components working together to improve student learning. Mosaic was designed as part of a larger assessment system, with the purpose of using assessment results to drive intervention and aim instruction at areas of need. Assessments can help schools drive universal interventions and targeted SEL initiatives. The Mosaic system provides individual student reports, following Hattie and Timperley’s (2007) recommendations for delivering formative feedback to students; reports inform students of their current levels on each skill, provide examples of the target skill level, and include recommendations on how students can improve. Mosaic also provides aggregate school-level reports so educators and administrators can be informed of broad areas of need in their schools and districts.

## 9. Limitations

This study was limited in a few ways. First, the students were not equally distributed across grades with an oversampling of 7th grade students in MS and 9th grade students in HS. This was an artefact of how schools opted to use Mosaic (i.e., to which students they administered Mosaic) and was not in our control. In addition, school-reported outcomes were limited. We relied on self-reported GPA, and the subsamples with school-reported discipline and attendance data were relatively small. Therefore, generalizations are limited. Additional data could be collected, particularly school-reported outcomes, to provide further validity evidence for the assessments. Moreover, while we focused primarily on the test-criterion validity evidence, additional validity evidence (e.g., construct validity) is also important. Assessment validation should be considered a continually ongoing process, with additional sources of evidence needed to strengthen the validity argument.

Another limitation was that we did not have access to participants’ SES or race/ethnicity status for most students. The exclusion of race/ethnicity data was not intentional, but rather a limitation induced from the assessment’s administration procedure during the 2018–2019 school year. During this administration window, schools were asked to provide students’ race/ethnicity information during the registration process. Reporting student race/ethnicity was optional, not required. We found that most schools chose not to provide students’ race/ethnicity information. Following that school year, we changed the assessment process to have students self-report their race/ethnicity during the test administration rather than relying on schools to do so during the registration process. This change in administration has resulted in more complete student race/ethnicity information beginning in the 2019–2020 school year.

However, we did have race/ethnicity data for 2% (*n* = 503) of the MS students and 18% (*n* = 1649) of the HS students, and we compared white students with underrepresented minority students. The groups scored significantly differently from one another on just one SE skill, Keeping an Open Mind, with the underrepresented students scoring higher in both MS and HS. We also note that race/ethnicity information was available during earlier phases of the assessment development, and the parameter estimates for scoring were extracted from a diverse and representative sample. Furthermore, we continue to gather assessment data complete with student-reported race/ethnicity information. With more recent assessment data (*N* = 3720), we have demonstrated that race/ethnicity subgroup differences are consistently small in magnitude across the three item types, suggesting fairness for all test-takers (Walton and Burrus 2022). DIF analyses across subgroups is also a component of our ongoing test validation process as our body of available assessment data continues to grow.

A final area for future work pertains to the discriminant validity issue raised above; that is, particularly in MS, there were several instances of weak discriminant validity. This could be due to a number of reasons. First, as discussed previously, SJT measures are often multidimensional. In line with this, the correlations involving SJT scores was typically where there were instances of weak discriminant validity. Second, we observed this more among the MS sample than the HS sample, and this is consistent with prior work showing that self-reports become more coherent within a skill domain and better differentiated across domains from late childhood to early adulthood (Soto et al. 2008). Soto and colleagues noted that the domains related to Getting Along with Others and Sustaining Effort showed large gains in the differentiation with age but small gains in coherence, and they conjectured that this differentiation could be the result of complex notions of right and wrong or good and bad. One possible implication of this pertains to impression management; that is, as children age, they should be more adept with impression management and able to present a coherent picture of a “good” respondent. This might suggest that the inclusion of the FC items in particular would be recommended for older students. Finally, this research (Soto et al. 2008) has shown that there is a high variability in the acquiescent responding among younger study participants. This may further explain the lack of differentiation among the younger students in the current sample and further point to the need for item types other than the Likert items for younger students.

## 10. Conclusions

Reliable and valid assessments are paramount for the growth of the field, as assessments enable practitioners to measure progress and maximize student SE skill development. Here, we argue that the assessment framework and response method are also important considerations for the development or selection of a SE skill assessment. Mosaic is one of a growing number of SE skills assessments designed to measure the skills that crosswalk to the Big Five personality framework. The current study offers additional support for this practice given that the skills accounted for a statistically significant amount of variance in important school-related constructs and outcomes. Moreover, the findings reported here highlight the importance of shying away from automatically using Likert-based assessments and considering more innovative item types either alone or in combination. Our findings suggest that the aggregate scores derived from multiple item types are often superior over single item types in terms of predictive validity, and that the single item type with the best validity evidence varies according to the outcome in question. It is often said that we measure what matters, and it is unequivocal that these five SE skills matter. By purposefully leveraging a multi-method SE skill assessment in schools, we can equip students with the skills they need to thrive in school, in the workforce, and beyond.

## Figures and Tables

**Table 1:** Alignment of Mosaic Skills to the Big Five Framework.

Big Five Factor	Mosaic Skill	Mosaic Skill Example Descriptors
Conscientiousness	Sustaining Effort	persistence, goal striving, reliability, attention to detail
Agreeableness	Getting Along with Others	collaboration, empathy, helpfulness, trustworthiness
Emotional Stability	Maintaining Composure	stress management, emotional regulation, poise
Openness	Keeping an Open Mind	creativity, inquisitiveness, open-mindedness, embracing diversity
Extraversion	Social Connection	assertiveness, influence, optimism, enthusiasm

**Table 2:** Multi-Trait Multi-Method Correlation Matrix.

Middle School	Likert					FC					SJT				
	SE	GA	MC	KO	SC	SE	GA	MC	KO	SC	SE	GA	MC	KO	SC
Likert															
SE															
GA	.53														
MC	.58	.63													
KO	.47	.60	.58												
SC	.39	.63	.52	.54											
FC															
SE	.43	.29	.31	.13	.20										
GA	.42	.49	.40	.33	.28	.35									
MC	.41	.31	.43	.22	.29	.53	.43								
KO	.24	.27	.26	.36	.30	.03	.42	.40							
SC	.27	.32	.26	.30	.48	.15	.27	.38	.52						
SJT															
SE	.55	.32	.31	.25	.16	.32	.36	.36	.23	.21					
GA	.44	.38	.28	.24	.12	.29	.43	.33	.25	.17	.63				
MC	.59	.54	.54	.43	.37	.37	.46	.41	.29	.29	.54	.52			
KO	.41	.48	.47	.55	.40	.14	.26	.21	.30	.27	.25	.23	.50		
SC	.45	.45	.38	.37	.39	.26	.34	.35	.31	.36	.49	.53	.55	.45	
*M* (*SD*)	4.41 (.67)	4.84 (.72)	4.37 (.77)	4.28 (.79)	4.46 (.71)	2.28 (.35)	2.46 (.38)	2.15 (.43)	2.13 (.37)	2.20 (.40)	3.24 (.63)	3.53 (.68)	3.54 (.55)	3.38 (.55)	3.41 (.52)
Cronbach’s Alpha	.71	.79	.74	.76	.72	.37	.50	.54	.35	.46	.76	.79	.63	.68	.59
**High School**	**Likert**					**FC**					**SJT**				
	**SE**	**GA**	**MC**	**KO**	**SC**	**SE**	**GA**	**MC**	**KO**	**SC**	**SE**	**GA**	**MC**	**KO**	**SC**
Likert															
SE															
GA	.55														
MC	.45	.53													
KO	.51	.58	.51												
SC	.45	.57	.44	.56											
FC															
SE	.38	.21	.23	.13	.17										
GA	.30	.50	.33	.32	.27	.24									
MC	.28	.24	.44	.27	.34	.49	.35								
KO	.08	.21	.20	.40	.37	−.10	.38	.35							
SC	.21	.23	.12	.30	.53	.10	.22	.39	.51						
SJT															
SE	.62	.47	.31	.42	.33	.28	.33	.22	.13	.18					
GA	.45	.57	.33	.45	.36	.23	.43	.24	.20	.19	.58				
MC	.28	.38	.30	.30	.26	.25	.36	.36	.22	.21	.40	.53			
KO	.34	.41	.29	.46	.27	.15	.36	.24	.29	.19	.46	.59	.49		
SC	.40	.40	.29	.41	.43	.17	.26	.25	.27	.28	.42	.45	.31	.42	
*M* (*SD*)	4.50 (.78)	4.94 (.61)	4.16 (.73)	4.54 (.68)	4.45 (.76)	2.34 (.35)	2.52 (.34)	2.20 (.43)	2.14 (.38)	2.23 (.43)	3.65 (.51)	3.65 (.48)	3.61 (.57)	3.41 (.52)	3.28 (.44)
Cronbach’s Alpha	.84	.77	.77	.76	.79	.40	.46	.58	.43	.57	.68	.63	.71	.65	.44

*Note.* SE = Sustaining Effort. GA = Getting Along with Others. MC = Maintaining Composure. KO = Keeping an Open Mind. SC = Social Connection.

**Table 3:** Associations Between Mosaic Skills and Outcome Variables: Correlations and Regression Statistics.

	Relationships with School Personnel			
	Likert	FC	SJT	Aggregate
Middle School ^a^				
Sustaining Effort	.47	.25	.32	.44
Getting Along with Others	.52	.37	.28	.49
Maintaining Composure	.54	.31	.47	.55
Keeping an Open Mind	.46	.22	.41	.47
Social Connection	.45	.28	.37	.47
*F* (5, 24,394)	2933.42 *	1119.52 *	1851.83 *	2690.51 *
*R* ^2^	.38	.19	.28	.36
High School ^b^				
Sustaining Effort	.41	.20	.32	.40
Getting Along with Others	.44	.29	.34	.44
Maintaining Composure	.37	.25	.28	.39
Keeping an Open Mind	.37	.15	.30	.36
Social Connection	.43	.21	.25	.38
*F* (5, 9106)	690.87 *	268.64 *	339.57 *	615.53 *
*R* ^2^	.28	.13	.16	.25
	**School Safety Climate**			
	**Likert**	**FC**	**SJT**	**Aggregate**
Middle School ^a^				
Sustaining Effort	.37	.21	.24	.35
Getting Along with Others	.42	.32	.23	.41
Maintaining Composure	.45	.24	.37	.44
Keeping an Open Mind	.32	.13	.29	.32
Social Connection	.28	.14	.25	.28
*F* (5, 24,394)	1548.96 *	660.76 *	903.88 *	1360.81 *
*R* ^2^	.24	.12	.16	.22
High School ^b^				
Sustaining Effort	.31	.16	.23	.30
Getting Along with Others	.34	.23	.25	.33
Maintaining Composure	.33	.17	.22	.32
Keeping an Open Mind	.22	.06	.20	.21
Social Connection	.20	.05	.12	.16
*F* (5, 24,394)	345.68 *	134.80 *	162.65 *	307.34 *
*R* ^2^	.16	.07	.08	.14
	**GPA**			
	**Likert**	**FC**	**SJT**	**Aggregate**
Middle School ^c^				
Sustaining Effort	.41	.24	.33	.41
Getting Along with Others	.21	.24	.26	.30
Maintaining Composure	.21	.26	.30	.32
Keeping an Open Mind	.17	.15	.15	.20
Social Connection	.17	.18	.25	.26
*F* (5, 22,777)	919.84 *	515.13 *	706.51 *	973.06 *
*R* ^2^	.17	.10	.13	.18
High School ^d^				
Sustaining Effort	.47	.28	.36	.47
Getting Along with Others	.23	.22	.28	.30
Maintaining Composure	.15	.23	.29	.29
Keeping an Open Mind	.20	.06	.23	.21
Social Connection	.12	.15	.17	.19
*F* (5, 8876)	542.49 *	232.46 *	322.70 *	521.24 *
*R* ^2^	.23	.12	.15	.23
	**Absences**			
	**Likert**	**FC**	**SJT**	**Aggregate**
Middle School ^e^				
Sustaining Effort	−.05	.03	−.08	−.04
Getting Along with Others	−.01	−.10	−.06	−.07
Maintaining Composure	.10	.01	−.03	.03
Keeping an Open Mind	.02	−.02	.08	.03
Social Connection	.07	−.03	.01	.02
*F* (5, 288)	1.78	.95	1.29	1.01
*R* ^2^	.03	.02	.02	.02
High School ^f^				
Sustaining Effort	−.14	−.11	−.14	−.17
Getting Along with Others	−.11	−.10	−.12	−.14
Maintaining Composure	−.06	−.07	−.12	−.11
Keeping an Open Mind	−.04	−.03	−.10	−.07
Social Connection	.02	−.04	−.06	−.04
*F* (5, 884)	6.66 *	3.30 *	4.39 *	6.14 *
*R* ^2^	.04	.02	.02	.03
	**Discipline**			
	**Likert**	**FC**	**SJT**	**Aggregate**
Middle School ^g^				
Sustaining Effort	−.15	−.11	−.20	−.20
Getting Along with Others	−.03	−.15	−.31	−.21
Maintaining Composure	−.09	−.05	−.11	−.11
Keeping an Open Mind	−.02	.07	.04	.04
Social Connection	.04	.09	−.11	.01
*F* (5, 344)	2.29 *	3.81 *	9.20 *	7.10 *
*R* ^2^	.03	.05	.12	.09
High School ^h^				
Sustaining Effort	−.01	−.03	−.13	−.08
Getting Along with Others	−.07	−.08	−.12	−.12
Maintaining Composure	−.07	−.01	−.14	−.10
Keeping an Open Mind	.01	.08	−.16	−.03
Social Connection	.10	.14	.02	.12
*F* (5, 715)	5.68 *	5.39 *	7.00 *	9.19 *
*R* ^2^	.04	.04	.05	.06

*Note.* ^a^*n* = 24,400. ^b^ *n* = 9,112. ^c^ *n* = 22,783. ^d^ *n* = 8,882. ^e^ *n* = 294. ^f^ *n* = 890. ^g^ *n* = 350. ^h^ *n* = 721. * *p* < .05.

**Table 4:** Mean Differences on Mosaic Skills Between Students with Acceptable, Habitual, and Chronic Absentee Records in High School Students.

	Acceptable ^a^		Habitual ^b^		Chronic ^c^			
	*M*	*SD*	*M*	*SD*	*M*	*SD*	*F*	*η* ^2^
Likert								
Sustaining Effort (SE)	4.50	.78	4.27	.81	4.12	.82	12.38 *	.03
Getting Along with Others (GA)	4.92	.61	4.90	.71	4.68	.73	5.26 *	.01
Maintaining Composure (MC)	4.17	.73	4.08	.70	4.03	.83	2.30	.01
Keeping an Open Mind (KO)	4.48	.71	4.40	.70	4.42	.70	1.04	.00
Social Connection (SC)	4.38	.76	4.48	.79	4.37	.82	1.26	.00
FC								
SE	2.34	.36	2.27	.35	2.24	.36	4.93 *	.01
GA	2.52	.33	2.47	.32	2.41	.38	4.52 *	.01
MC	2.21	.44	2.14	.43	2.12	.41	2.71	.01
KO	2.13	.41	2.16	.37	2.10	.38	.67	.00
SC	2.25	.44	2.25	.44	2.17	.42	1.32	.00
SJT								
SE	3.63	.51	3.52	.46	3.47	.47	6.71 *	.02
GA	3.62	.48	3.60	.45	3.41	.47	7.25 *	.02
MC	3.61	.56	3.47	.57	3.38	.57	9.08 *	.02
KO	3.38	.52	3.32	.48	3.22	.47	3.91 *	.01
SC	3.28	.45	3.30	.40	3.15	.45	3.62 *	.01
Aggregate								
SE	.00	2.36	−.74	2.17	−1.08	2.24	13.05 *	.03
GA	−.12	2.39	−.33	2.33	−1.25	2.59	8.40 *	.02
MC	.04	2.29	−.50	2.10	−.77	2.12	7.69 *	.02
KO	−.20	2.39	−.37	2.16	−.69	2.04	1.86	.00
SC	−.09	2.40	.08	2.25	−.58	2.31	2.23	.01

*Note.*^a^*n* = 636. ^b^ *n* = 168. ^c^ *n* = 86. *F* df = 2, 887. * *p* < .05.

**Table 5:** Mean Differences on Mosaic Skills Between Students with None and Some Discipline Infractions.

Middle School	0 Infractions ^a^		>0 Infractions ^b^			
	*M*	*SD*	*M*	*SD*	*t*	*d*
Likert						
Sustaining Effort (SE)	4.49	.63	4.25	.59	4.25 *	.40
Getting Along with Others (GA)	4.87	.67	4.68	.67	3.09 *	.29
Maintaining Composure (MC)	4.38	.68	4.18	.77	3.09 *	.29
Keeping an Open Mind (KO)	4.27	.78	4.05	.81	2.93 *	.28
Social Connection (SC)	4.42	.69	4.35	.66	1.14	.11
FC						
SE	2.34	.33	2.23	.32	3.45 *	.33
GA	2.53	.33	2.40	.38	4.10 *	.39
MC	2.18	.44	2.12	.43	1.65 *	.16
KO	2.12	.38	2.13	.41	−.20	.02
SC	2.22	.42	2.22	.40	−.11	.01
SJT						
SE	3.39	.63	3.07	.54	5.56 *	.52
GA	3.72	.59	3.41	.62	5.51 *	.52
MC	3.64	.54	3.42	.54	4.16 *	.39
KO	3.41	.56	3.26	.57	2.82 *	.27
SC	3.49	.54	3.32	.52	3.39 *	.32
Aggregate						
SE	.41	2.31	−.82	2.08	5.82 *	.55
GA	.39	2.28	−.78	2.36	5.40 *	.51
MC	.28	2.26	−.55	2.41	3.81 *	.36
KO	.17	2.35	−.35	2.36	2.34 *	.22
SC	.14	2.40	−.27	2.24	1.87 *	.18
**High School**	**0 Infractions ^c^**		**>0 Infractions ^d^**			
	***M***	***SD***	***M***	***SD***	***t***	***d***
Likert						
SE	4.56	.75	4.46	.78	2.36 *	.13
GA	4.97	.57	4.88	.66	2.80 *	.15
MC	4.21	.70	4.10	.77	2.93 *	.16
KO	4.59	.66	4.56	.70	1.05	.06
SC	4.36	.75	4.60	.76	−5.59 *	.31
FC						
SE	2.34	.35	2.34	.35	.21	.01
GA	2.55	.33	2.49	.33	3.81 *	.21
MC	2.22	.44	2.20	.42	.79	.04
KO	2.16	.39	2.18	.38	−.62	.03
SC	2.21	.45	2.30	.39	−3.74 *	.20
SJT						
SE	3.72	.49	3.60	.53	4.62 *	.25
GA	3.73	.47	3.63	.48	3.78 *	.21
MC	3.69	.53	3.57	.58	4.02 *	.22
KO	3.50	.51	3.33	.50	6.05 *	.33
SC	3.32	.47	3.29	.44	1.25	.07
Aggregate						
SE	.26	2.27	−.12	2.29	3.11 *	.17
GA	.31	2.30	−.25	2.40	4.33 *	.24
MC	.26	2.25	−.16	2.20	3.43 *	.19
KO	.27	2.33	−.08	2.22	2.79 *	.15
SC	−.13	2.44	.31	2.19	−3.39 *	.19

*Note*. ^a^*n* = 335. ^b^ *n* = 169. ^c^ *n* = 982. ^d^ *n* = 508. * *p* < .05.

## Data Availability

The data presented in this study are available on request from the corresponding author. The data are not publicly available due to contractual and privacy issues.

## References

[B1-jintelligence-10-00072] Abrahams Loes, Pancorbo Gina, Primi Ricardo, Santos Daniel, Kyllonen Patrick, John Oliver P., De Fruyt Filip (2019). Social-emotional skill assessment in children and adolescents: Advances and challenges in personality, clinical, and educational contexts. Psychological Assessment.

[B2-jintelligence-10-00072] ACT (2016). Development and Validation of ACT Engage^®^: Technical Manual.

[B3-jintelligence-10-00072] ACT (2021). Mosaic by ACT^®^: Social Emotional Learning Assessment Technical Manual.

[B4-jintelligence-10-00072] Allen Jeff, Way Jason D., Casillas Alex (2019). Relating school context to measures of psychosocial factors for students in grades 6 through 9. Personality and Individual Differences.

[B5-jintelligence-10-00072] Aspen Institute (2018). From a Nation at Risk to a Nation at Hope: Recommendations from the National Commission on Social, Emotional, and Academic Development.

[B6-jintelligence-10-00072] Association of American Medical Colleges (2022). AAMC PREview^TM^ Professional Readiness Exam Now Available for 2023 Admissions Cycle. https://students-residents.aamc.org/aamc-preview/aamc-preview-professional-readiness-exam-now-available-2023-admissions-cycle.

[B7-jintelligence-10-00072] Attendance Works Chronic Absence. https://www.attendanceworks.org/chronic-absence/the-problem/.

[B8-jintelligence-10-00072] Barrick Murray R., Mount Michael K., Judge Timothy A. (2001). Personality and performance at the beginning of the new millennium: What do we know and where do we go next?. International Journal of Selection and Assessment.

[B9-jintelligence-10-00072] Berg Juliette, Osher David, Same Michelle R., Nolan Elizabeth, Benson Deaweh, Jacobs Naomi (2017). Identifying, Defining, and Measuring Social and Emotional Competencies. American Institutes for Research.

[B10-jintelligence-10-00072] Black Paul, Wiliam Dylan (2009). Developing the theory of formative assessment. Educational Assessment, Evaluation and Accountability.

[B11-jintelligence-10-00072] Bogg Tim, Roberts Brent W. (2004). Conscientiousness and health-related behaviors: A meta-analysis of the leading behavioral contributors to mortality. Psychological Bulletin.

[B12-jintelligence-10-00072] Burrus Jeremy, Mattern Krista, Naemi Bobby D., Roberts Richard D. (2017). Building Better Students: Preparation for the Workforce.

[B13-jintelligence-10-00072] Cao Mengyang, Drasgow Fritz (2019). Does forcing reduce faking? A meta-analytic review of forced-choice personality measures in high-stakes situations. Journal of Applied Psychology.

[B14-jintelligence-10-00072] Casillas Alex, Way Jason, Burrus Jeremy, O’Connor Ryan, Camara Wayne, Mattern Krista, Hanson Mary Ann (2015). Behavioral skills. Beyond Academics: A Holistic Framework for Enhancing Education and Workplace Success.

[B15-jintelligence-10-00072] Cattell Raymond B. (1943). The description of personality: The foundations of trait measurement. Psychological Review.

[B16-jintelligence-10-00072] Chamorro-Premuzic Tomas, Furnham Adrian (2003). Personality predicts academic performance: Evidence from two longitudinal university samples. Journal of Research in Personality.

[B17-jintelligence-10-00072] Chernyshenko Oleksandr S., Kankaraš Miloš, Drasgow Fritz (2018). Social and Emotional Skills for Student Success and Wellbeing: Conceptual Framework for the OECD Study on Social and Emotional Skills.

[B18-jintelligence-10-00072] Christiansen Neil D., Burns Gary N., Montgomery George E. (2005). Reconsidering forced-choice item formats for applicant personality assessment. Human Performance.

[B19-jintelligence-10-00072] Colorado Department of Education Student Attendance. http://www.cde.state.co.us/communications/schoolattendance-factsheet-2018.

[B20-jintelligence-10-00072] Corcoran Roisin P., Cheung Alan C. K., Kim Elizabeth, Xie Chen (2018). Effective universal school-based social and emotional learning programs for improving academic achievement: A systematic review and meta-analysis of 50 years of research. Educational Research Review.

[B21-jintelligence-10-00072] Credé Marcus, Roch Sylvia G., Kieszczynka Urszula M. (2010). Class attendance in college: A meta-analytic review of the relationship of class attendance with grades and student characteristics. Review of Educational Research.

[B22-jintelligence-10-00072] Davidson Laura A., Crowder Marisa K., Gordon Rachel A., Domitrovich Celene E., Brown Randal D., Hayes Benjamin I. (2018). A continuous improvement approach to social and emotional competency measurement. Journal of Applied Developmental Psychology.

[B23-jintelligence-10-00072] de Raad Boele, Mlačić Boris, Widiger Thomas A. (2015). The lexical foundation of the Big Five-Factor Model. The Oxford handbook of the Five Factor Model.

[B24-jintelligence-10-00072] Denham Susanne A., Durlak Joseph A., Domitrovich Celene E., Weissberg Roger P., Gullotta Thomas P. (2015). Assessment of SEL in educational contexts. Handbook of Social and Emotional Learning: Research and Practice.

[B25-jintelligence-10-00072] Durlak Joseph A., Domitrovich Celene E., Weissberg Roger P., Gullotta Thomas. P. (2015). Handbook of Social and Emotional Learning: Research and Practice.

[B26-jintelligence-10-00072] Goldberg Lewis R. (1993). The structure of phenotypic personality traits. American Psychologist.

[B27-jintelligence-10-00072] Hattie John, Timperley Helen (2007). The power of feedback. Review of Educational Research.

[B28-jintelligence-10-00072] Hontangas Pedro M., De La Torre Jimmy, Ponsoda Vicente, Leenen Iwin, Morillo Daniel, Abad Francisco J. (2015). Comparing traditional and IRT scoring of forced-choice tests. Applied Psychological Measurement.

[B29-jintelligence-10-00072] Hooper Amy C., Cullen Michael J., Sackett Paul R., Weekley Jeff A., Ployhart Robert E. (2006). Operational threats to the use of Situational Judgment Tests: Faking, coaching, and retesting issues. Situational Judgment Tests.

[B30-jintelligence-10-00072] Hudson Nathan W., Briley Daniel A., Chopik William J., Derringer Jaime (2019). You have to follow through: Attaining behavioral change goals predicts volitional personality change. Journal of Personality and Social Psychology.

[B31-jintelligence-10-00072] Jackson Douglas N., Wroblewski Victor R., Ashton Michael C. (2000). The impact of faking on employment tests: Does forced choice offer a solution?. Human Performance.

[B32-jintelligence-10-00072] Jones Stephanie, Bailey Rebecca, Brush Katharine, Nelson Bryan, Barnes Sophie (2016). What Is the Same and What Is Different? Making Sense of the “Noncognitive” Domain: Helping Educators Translate Research into Practice. Harvard Graduate School of Education. https://easel.gse.harvard.edu/files/gse-easel-lab/files/words_matter_paper.pdf.

[B33-jintelligence-10-00072] Kankaraš Miloš, Suarez-Alvarez Javier (2019). Assessment Framework of the OECD Study on Social and Emotional Skills. https://www.oecd-ilibrary.org/education/assessment-framework-ofthe-oecd-study-on-social-and-emotional-skills_5007adef-en.

[B34-jintelligence-10-00072] Kankaraš Miloš, Feron Eva, Renbarger Rachel (2019). Assessing Students’ Social and Emotional Skills through Triangulation of Assessment Methods.

[B35-jintelligence-10-00072] Kautz Tim, Heckman James J., Diris Ron, Weel Bas ter, Borghans Lex (2014). Fostering and measuring skills: Improving noncognitive skills to promote lifetime success. OECD, Educational and Social Progress.

[B36-jintelligence-10-00072] Kenny David A., Kashy Deborah A. (1992). Analysis of the multitrait-multimethod matrix by confirmatory factor analysis. Psychological Bulletin.

[B37-jintelligence-10-00072] LeBuffe Paul A., Shapiro Valerie B., Robitaille Jennifer L. (2018). The Devereux Student Strengths Assessment (DESSA) comprehensive system: Screening, assessing, planning, and monitoring. Journal of Applied Developmental Psychology.

[B38-jintelligence-10-00072] Lievens Filip, Peeters Helga, Schollaert Eveline (2008). Situational judgment tests: A review of recent research. Personnel Review.

[B39-jintelligence-10-00072] Lipnevich Anastasiya A., MacCann Carolyn, Roberts Richard D., Saklofske Donald H., Reynolds Cecil R., Schwean Vicki (2013). Assessing noncognitive constructs in education: A review of traditional and innovative approaches. Oxford Handbook of Child Psychological Assessment.

[B40-jintelligence-10-00072] Lounsbury John W., Steel Robert P., Loveland James M., Gibson Lucy W. (2004). An investigation of personality traits in relation to adolescent school absenteeism. Journal of Youth and Adolescence.

[B41-jintelligence-10-00072] Mahoney Joseph L., Durlak Joseph A., Weissberg Roger P. (2018). An update on social and emotional learning outcome research. Phi Delta Kappan.

[B42-jintelligence-10-00072] Mammadov Sakhavat (2021). Big Five personality traits and academic performance: A meta-analysis. Journal of Personality.

[B43-jintelligence-10-00072] Marsh Herbert W., Hau Kit-Tai (2003). Big-fish--little-pond effect on academic self-concept: A cross-cultural (26-country) test of the negative effects of academically selective schools. American Psychologist.

[B44-jintelligence-10-00072] Martin Jonathan E., Menten Alexis, Walton Kate E., Burrus Jeremy, Anguiano-Carrasco Cristina, Olaru Gabriel, Roberts Richard D. (2019). A Rosetta Stone for Social and Emotional Skills.

[B45-jintelligence-10-00072] McCrae Robert R., Terracciano Antonio, Members of the Personality Profiles of Cultures Project (2005). Universal features of personality traits from the observer’s perspective: Data from 50 cultures. Journal of Personality and Social Psychology.

[B46-jintelligence-10-00072] Meade Adam W. (2004). Psychometric properties and issues involved with creating and using ipsative measures for selection. Journal of Occupational and Organizational Psychology.

[B47-jintelligence-10-00072] Organisation for Economic Co-Operation and Development (2015). Skills for Social Progress: The Power of Social and Emotional Skills.

[B48-jintelligence-10-00072] Osher David, Berg Juliette (2017). School Climate and Social and Emotional Learning: The Integration of Two Approaches.

[B49-jintelligence-10-00072] Poropat Arthur E. (2009). A meta-analysis of the five-factor model of personality and academic performance. Psychological Bulletin.

[B50-jintelligence-10-00072] Roberts Brent W., Walton Kate E., Viechtbauer Wolfgang (2006). Patterns of mean-level change in personality traits across the life course: A meta-analysis of longitudinal studies. Psychological Bulletin.

[B51-jintelligence-10-00072] Roberts Brent W., Luo Jing, Briley Daniel A., Chow Philip I., Su Rong, Hill Patrick L. (2017). A systematic review of personality trait change through intervention. Psychological Bulletin.

[B52-jintelligence-10-00072] Schmitt David P., Allik Jüri, McCrae Robert R., Benet-Martínez Verónica (2007). The geographic distribution of Big Five personality traits: Patterns and profiles of human self–description across 56 nations. Journal of Cross-Cultural Psychology.

[B53-jintelligence-10-00072] Soto Christopher J., John Oliver P., Gosling Samuel D., Potter Jeff (2008). The developmental psychometrics of the Big Five self-reports: Acquiescence, factor structure, coherence, and differentiation from ages 10 to 20. Journal of Personality and Social Psychology.

[B54-jintelligence-10-00072] Soto Christopher J., Napolitano Christopher M., Sewell Madison N., Yoon Hee J., Roberts Brent W. (2022). An integrative framework for conceptualizing and assessing social, emotional, and behavioral skills: The BESSI. Journal of Personality and Social Psychology.

[B55-jintelligence-10-00072] Tackett Jennifer L. (2006). Evaluating models of the personality-psychopathology relationship in children and adolescents. Clinical Psychology Review.

[B56-jintelligence-10-00072] Tackett Jennifer L., Kushner Shauna C., De Fruyt Filip, Mervielde Ivan (2013). Delineating personality traits in childhood and adolescence: Associations among measures, temperament, and behavioral problems. Assessment.

[B57-jintelligence-10-00072] Tupes Ernest C., Christal Raymond E. (1961). Recurrent Personality Factors Based on Trait Ratings.

[B58-jintelligence-10-00072] Walton Kate E., Burrus Jeremy (2022). Mosaic™ by ACT^®^ Social Emotional Learning Assessment: Evaluating Racial Subgroup Differences Across Item Types.

[B59-jintelligence-10-00072] Walton Kate E., Murano Dana, Burrus Jeremy, Casillas Alex (2021). Multi-method support for using the Big Five framework to organize social and emotional skills. Assessment.

[B60-jintelligence-10-00072] Whetzel Deborah L., McDaniel Michael A. (2009). Situational judgment tests: An overview of current research. Human Resource Management Review.

[B61-jintelligence-10-00072] Whetzel Deborah L., McDaniel Michael A., Kumar Updesh (2016). Are situational judgment tests better assessments of personality than traditional personality tests in high-stakes testing?. The Wiley Handbook of Personality Assessment.

[B62-jintelligence-10-00072] Zell Ethan, Lesick Tara L. (2021). Big five personality traits and performance: A quantitative synthesis of 50+ meta-analyses. Journal of Personality.

[B63-jintelligence-10-00072] Zickar Michael J., Gibby Robert E., Robie Chet (2004). Uncovering faking samples in applicant, incumbent, and experimental data sets: An application of mixed-model item response theory. Organizational Research Methods.

